# The RabGEF ALS2 is a hypoxia inducible target associated with the acquisition of aggressive traits in tumor cells

**DOI:** 10.1038/s41598-020-79270-6

**Published:** 2020-12-18

**Authors:** Solange Rivas, Patricio Silva, Montserrat Reyes, Hugo Sepúlveda, Luis Solano, Juan Acuña, Marisol Guerrero, Manuel Varas-Godoy, Andrew F. G. Quest, Martín Montecino, Vicente A. Torres

**Affiliations:** 1 Institute for Research in Dental Sciences, Faculty of Dentistry, Universidad de Chile, Calle Sergio Livingstone 943, Independencia, Santiago Chile; 2 Advanced Center for Chronic Diseases (ACCDiS), Universidad de Chile, Santiago, Chile; 3 Department of Pathology and Oral Medicine, Faculty of Dentistry, Universidad de Chile, Santiago, Chile; 4 Institute of Biomedical Sciences and FONDAP Center for Genome Regulation, Faculty of Medicine and Faculty of Life Sciences, Universidad Andrés Bello, Santiago, Chile; 5 Laboratory of Pathological Anatomy, Hospital San José, Santiago, Chile; 6 Center for Cell Biology and Biomedicine (CEBICEM), Faculty of Medicine and Science, Universidad San Sebastián, Santiago, Chile; 7 Center for Studies on Exercise, Metabolism and Cancer (CEMC), Biomedical Sciences Institute (ICBM), Faculty of Medicine, Universidad de Chile, Santiago, Chile

**Keywords:** Cancer microenvironment, Cell biology

## Abstract

Tumor hypoxia and the hypoxia inducible factor-1, HIF-1, play critical roles in cancer progression and metastasis. We previously showed that hypoxia activates the endosomal GTPase Rab5, leading to tumor cell migration and invasion, and that these events do not involve changes in Rab protein expression, suggesting the participation of intermediate activators. Here, we identified ALS2, a guanine nucleotide exchange factor that is upregulated in cancer, as responsible for increased Rab5-GTP loading, cell migration and metastasis in hypoxia. Specifically, hypoxia augmented ALS2 mRNA and protein levels, and these events involved HIF-1α-dependent transcription, as shown by RNAi, pharmacological inhibition, chromatin immunoprecipitation and bioinformatics analyses, which identified a functional HIF-1α-binding site in the proximal promoter region of ALS2. Moreover, ALS2 and Rab5 activity were elevated both in a model of endogenous HIF-1α stabilization (renal cell carcinoma) and by following expression of stable non-hydroxylatable HIF-1α. Strikingly, ALS2 upregulation in hypoxia was required for Rab5 activation, tumor cell migration and invasion, as well as experimental metastasis in C57BL/6 mice. Finally, immunohistochemical analyses in patient biopsies with renal cell carcinoma showed that elevated HIF-1α correlates with increased ALS2 expression. Hence, this study identifies ALS2 as a novel hypoxia-inducible gene associated with tumor progression and metastasis.

## Introduction

Hypoxia is a common condition of the tumor microenvironment, which develops as a consequence of uncontrolled and rapid grow of solid tumors, along with aberrant angiogenesis^1^. Pressure conditions imposed by hypoxia lead tumor cells to adapt by re-programming cellular metabolism, gene expression, protein homeostasis and intracellular trafficking^1–3^. Thus, hypoxia has been associated with poor prognosis and increased risk of metastasis, and it is known to stimulate tumor cell migration and local invasion by mechanisms that are not completely understood^1,4^.

Most adaptative responses in hypoxia involve stabilization of hypoxia inducible factors (HIFs), amongst which HIF-1 is the most studied, and has been directly associated with tumor cell metastasis^5^. HIF-1 is a heterodimeric transcription factor, composed of the constitutively expressed subunit HIF-1β, and the oxygen-sensitive subunit HIF-1α. HIF-1α is constitutively degraded by the action of prolyl-hydroxylases, which hydroxylate HIF-1α on P402 and P564, allowing recognition by the tumor suppressor protein von Hippel Lindau (VHL), an ubiquitin ligase that targets HIF-1α for proteasome-mediated degradation. Conversely, in hypoxia, HIF-1α is no longer degraded, allowing its accumulation and subsequent heterodimerization with HIF-1β, to induce the transcription of target genes via binding to a conserved consensus sequence 5′-[A/G]CGTG-3′^5,6^. Although most evidence points to a direct effect of HIF-1α on the expression of target genes, some studies have shown an intriguing connection between hypoxia and the endocytic machinery via different mechanisms^3,7–9^. Specifically, early reports showed that hypoxia affects the dynamics of two endosomal populations, early endosomes via repression of the endosomal effector protein Rabaptin5^3^, and recycling endosomes via re-localization of the small GTPase Rab11^9^. In addition, previous studies by our group showed that hypoxia activates the early endocytic protein, Rab5, thereby allowing Rac1 activation, lamellipodia formation, tumor cell migration, invasion and metastasis^7^. This was intriguing, because unlike the reported effects on hypoxia-induced transcription of another small GTPase, RhoA^10^, no evidence is available linking HIF1 to alterations in small GTPase activity. However, our reported findings indicate that hypoxia stimulates Rab5 GTP-loading, without any fluctuations in total Rab5 expression, and that these events appear to be adaptative and sustained in time, since re-oxygenation did not diminish elevated Rab5-GTP levels, and RNAi-mediated knockdown of HIF-1α decreased hypoxia-driven Rab5 activation^7^. In conjunction, these observations suggest that intermediate, currently unknown factors are involved in Rab5 activation by hypoxia.

Rab5 activity is controlled by guanine nucleotide exchange factors (GEF) and GTPase activating proteins (GAP), which activate (increasing Rab5-GTP levels) and inactivate Rab5 (promoting Rab5-GTP hydrolysis), respectively^11^. In this respect, RabGEFs have gained attention in cancer research, as some of them have been related with malignancy and tumor progression. Specifically, elevated RIN1 expression is associated with tumor grade and lymph node metastasis in melanoma^12^, and it was found to promote renal cell carcinoma malignancy^13^. Alternatively, RIN3 is upregulated in T-cell large granular lymphocyte leukemia^14^. Likewise, Rabex-5 is upregulated in gastric cancer, small and non-small cell lung cancer, and in colorectal cancer^15–18^. Beyond these data, little is known in this context about other Rab5GEFs, although recent studies indicate that ALS2-mediated activation of Rab5 at the mitochondria prevents oxidative stress-induced cytotoxicity in cervicouterine cancer cells^19^. Most importantly, no Rab protein regulators, and particularly Rab5GEFs, have been shown to be sensitive to hypoxia.

Since Rab5 is a critical regulator of hypoxia-induced cell migration and metastasis, and Rab5 activity, but not total Rab5 expression, is increased by hypoxia in a HIF-1α-dependent manner, we hypothesized that intermediate, yet unknown factors, are involved in such events. Here, we identified the Rab5GEF ALS2 as a novel HIF-1α target that accounts for increased Rab5-GTP loading, tumor cell migration and invasion in hypoxia. Importantly, increased ALS2 expression correlates with augmented HIF-1α levels in cancer biopsies, making it a promising marker for the detection of HIF-1α and hypoxia-related cancers.

## Materials and methods

### Materials

Monoclonal anti-Rab5 (sc46692) and anti-RhoA (sc-418) were from Santa Cruz Biotechnologies. Other antibodies included monoclonal anti-ALS2 (ab170896, Abcam), anti-HIF1α (#610959, BD Transduction Laboratories) and ChIP-grade polyclonal anti-HIF-1α (ab2185, Abcam). Goat anti-rabbit and goat anti-mouse antibodies coupled to horseradish peroxidase and anti-actin antibody (#A5316, SIGMA) were from Bio-Rad Laboratories (Hercules, CA). Tissue culture medium, antibiotics and fetal bovine serum were from GIBCO Life Technologies (Grand Island, NY) and HyClone Laboratories (Logan, UT). Glutathione-Sepharose 4B was from GE Healthcare (Piscataway, NJ). The EZ-ECL chemiluminescent substrate was from Pierce Chemical (Rockford, IL). The HIF-1α inhibitor (10uM, sc-205346) and siRNA constructs targeting HIF-1α (mix of 3 different siRNAs targeting human HIF-1α, sc-35561), ALS2 (mix of 3 different siRNAs targeting human ALS2, sc-60154) and siRNA control (sc-37007), were from Santa Cruz Biotechnology. Transfections were performed with Optimem (#31985088, Gibco), the Lipofectamine 2000 (#11668027) and the Lipofectamine RNAiMAX (13778075), both from Invitrogen.

### Plasmids

The pEGFP-C1 plasmids encoding wild type Rab5, Rab5/S34N and Rab5/Q79L were previously described^20,21^. The pEGFP-C1 plasmids encoding wild type ALS2 and VPS-deficient ALS2 (GFP-ALS2^ΔVPS^) were kindly donated by Dr. Justin Topp^22^. The pcDNA3.1 plasmid encoding for wild type HIF-1α and stable, non-hydroxylatable HIF-1α^P564A/P402A^ were previously described and kindly donated by Dr. Salvador Moncada^23^.

### Cell culture

A549 lung carcinoma cells (ATCC, CCL-185), B16-F10 murine melanoma (ATCC, CRL-6475), RCC4 human clear cell renal cell carcinoma (ECACC 03112702) and von Hippel Lindau-reconstituted RCC4 (RCC4^+vhl^, ECACC 03112703) were maintained in standard cell culture conditions (37ºC, 5% CO_2_). A549, RCC4 and RCC4^+vhl^ were cultured in DMEM high glucose, supplemented with 10% fetal bovine serum and antibiotics (penicillin 10.000 U/ml; streptomycin 10 μg/ml). B16-F10 cells were cultured in RPMI medium supplemented with 10% fetal bovine serum and antibiotics. Hypoxia was performed as previously described, using a hermetically sealed, modular incubator chamber (MIC-101 Billups-Rothenberg Inc., Del Mar, CA), and a gas mixture (1% O2, 5% CO2 and 94% N2, Linde Group)^7^.

### Pulldown assay

Rab5-GTP levels were measured as previously described^21,24^. Cells were lysed in a buffer containing 25 mM HEPES (pH 7.4), 100 mM NaCl, 5 mM MgCl_2_, 1% NP 40, 10% glycerol, 1 mM dithiothreitol and protease inhibitors. Extracts were incubated for 5 min on ice and clarified by centrifugation (10,000×*g*, 1 min, 4 °C). Post-nuclear supernatants were used for pulldown assays with GSH beads precoated with GST-R5BD. Incubations were allowed for 15 min at 4 °C in a rotating shaker, beads were collected, washed and analyzed by Western blot. Of note, homogenates prepared for pulldown analysis are not appropriate for total protein quantification and uneven loading is possible.

### Western blots

Total protein extracts were resolved by SDS-PAGE, transferred to nitrocellulose membranes, blocked with 5% gelatin in 0.1% TBS-Tween20, and blotted with specific antibodies, as indicated. Primary antibodies were prepared in blocking solution and membranes were incubated all night at 4 °C, washed in TBS-Tween20 and further incubated with HRP-conjugated secondary antibodies. Samples were detected with EZ-ECL and visualized in an Amersham Imager 600 (GE Healthcare).

### In silico analysis of putative HIF-1α target genes

Complete gene sequences of different Rab5GEFs were obtained from http://www.ensembl.org/Homo_sapiens/Gene/ database (RIN1, ENSG00000174791; RIN2, ENSG00000132669; RIN3, ENSG00000100599; RABEX-5, ENSG00000154710; ALS2, ENSG00000003393), classified with respect to their nucleotide sequence as ‘proximal promoter region’ (up to − 2000 bp) or ‘coding sequence’ (+ 1 site), and putative consensus HREs (5′-[A/G]CGTG-3´) were screened within those sequences (Supplementary Fig. 1A).

### RNA extraction and analysis

Total RNA was extracted with TRIZOL (Invitrogen, Life Techonogies). For cDNA synthesis, samples were treated with RNase-Free DNase kit (#M6101 Promega), quantified and purity was verified for subsequent reverse transcription, using the cDNA Reverse Transcription Kit (Applied Biosystems). VEGFA, actin and the different Rab5GEFs were quantified by qRT-PCR (Applied Biosystems, ΔΔCt method), using the following forward and reverse primers, respectively: VEGFA, 5′-CTCTACCTCCACCATGCCAAG-3′, 5′-AGACATCCATGAACTTCACCACTT-3′; actin, 5′-TGGCACCCAGCACAATGAAGA-3′, 5′-GAAGCATTTGCGGTGGACGAT-3′; RIN1, 5′-GCCTTGCCTTGGGACTGGAT-3′, 5′-AGTAGTGAAGCTGGACGGGCT-3′; RIN2, 5′-TTGCCTTCCCGCTTCTACATGC-3′,5′-TAAAGTTGCCCTTGGCCGAGTT-3′; RIN3, 5′-TGCCTTCGGGACCCTCACT-3′, 5′-AGCCAGCATGTTGACGGACAC-3′; ALS2, 5′-TCGCCTAAAGGATGCCACCTATGA-3′, 5′-TCTCCATACCCATCTTCCAAGCCA-3′; RABEX-5 5′-TGTGCTGTGCTGTGGCTTTCAT-3′, 5′-GCTTGACGCCTAAGCAAGCATC-3’.

### Chromatin immunoprecipitation (ChIP)

Samples were fixed for 10 min in 1% formaldehyde in PBS (# 28906, Thermo Scientific), and fixation was stopped with 0.125 M glycine. Then, cells were pelleted at 5000 rpm, 4 °C, for 5 min, resuspended in lysis buffer containing 50 mM HEPES (pH 7.8), 3 mM MgCl_2_, 20 mM KCl, 0.1% NP-40 and protease inhibitors, and homogenized with a glass pistil via 60 strokes. Samples were then centrifuged and resuspended in sonication buffer containing 50 mM HEPES (pH 7.8), 140 mM NaCl, 1 mM EDTA, 1% Triton X-100, 0.1% sodium deoxycholate, 0.1% SDS and protease inhibitors. Chromatin fragmentation was performed by sonication, generating fragments of 200–500 bp. Samples were incubated with proteinase K (60 min, 50 °C) and precleared with a non-related IgG. HIF-1α was immunoprecipitated overnight with a ChIP grade, primary antibody, and DNA/protein/antibody complexes were captured by adding protein A/G. DNA purification was carried out with phenol/chloroform:isoamylalcohol (1:27) and the enrichment of HIF-1α with specific DNA fragments was measured by qPCR, using the following forward and reverse primers, respectively: RhoA, 5′-CCTATCCTACAGGCTGCTGAA-3′, 5′-TAAGCCCACCAGCTTAATGG-3′; ALS2, 5′-GACTGTCTTAGGCTCAGCAATA-3′, 5′-TTCACCAACACCCTCAACTC-3′; actin 5′-CGGCCCAAAGGACTTTTA-3′, 5′-TTCCCCTGACTCAGCCTTT-3’.

### Migration and invasion assays

Cell migration and invasion were evaluated in Boyden Chamber and Matrigel assays, as previously reported^7^.

### Syngenic lung colonization assay

This model of experimental metastasis was previously described by us and others and is based on lung colonization by metastatic cells once injected into the tail vein of mice^7,25^. Lung colonization by B16-F10 cells proceeds with rapid kinetics and is defined by events that occur within a few hours post-injection^26^. Specifically, it was previously shown that after 24 h of exposure to hypoxia, B16-F10 mouse melanoma cells significantly increase their ability to colonize the lung^7,25^. Here, B16-F10 cells were transfected with either siRNA-control or siRNA-ALS2, simultaneously exposed to normoxia or hypoxia for 24 h and then injected intravenously into the tail vein of C57BL/6 mice (2 × 10^5^ cells). Mice were then sacrificed after 21 days, and lungs were fixed in Feketes solution. Black tumor masses were separated from the rest of the lung and weighed. Metastasis (lung colonization) was expressed as black tissue mass/total lung mass (%). Experimental protocols were approved by the institutional bioethics committee (Protocol CBA1053, Faculty of Medicine, Universidad de Chile).

### Immunohistochemical analysis

Biopsies diagnosed with clear cell renal cell carcinoma and tumor-free areas in peritumoral region were obtained with the approval of the Research Ethics Committee (North Metropolitan Health Service, Santiago, Chile). Tumor and tumor-free regions were validated by the Pathological Anatomy Service, Hospital San José, Santiago, Chile. Sections of 3 µm thickness were obtained with a microtome. For antigen recovery post-fixation, sections were treated with sodium citrate (pH 6.0) for 45 min, and endogenous peroxidase was blocked by incubation with 3% H_2_O_2_ in methanol at room temperature, for 20 min. Sections were incubated in horse serum for 30 min at room temperature, and subsequently incubated with the primary antibodies for 1 h at 37 °C. Immunostaining with primary antibodies was previously standardized for optimal concentrations and incubation conditions, using clear cell renal cell carcinoma and oral squamous cell carcinoma biopsies. As controls healthy renal tissue and healthy oral mucosa, were employed (for details, see Supplementary Fig. 2). Biotinylated second antibodies were incubated for 30 min at 37 °C, and finally incubated with peroxidase-streptavidin conjugates (Vectastain Elite Universal Detection System Kit ABC-HRP, RTU, Vector-USA, USA) for 30 min at 37 °C. The reaction was visualized with diaminobenzidine and contrasted with Harry´s hematoxylin.

### Statistical analysis

All statistical analyses were performed with at least 3 independent experiments. Statistical differences between 2 conditions were assessed with non-parametric t test (Mann–Whitney correction). Comparisons between more than 2 conditions were analyzed with non-parametric one-way ANOVA (Kruskall-Wallis correction). Analyzes were performed with the GraphPad Prism 6 Software (San Diego, CA).

### Ethics approval

All methods were carried out in accordance with relevant guidelines and regulations.

## Results

### HIF-1α activates Rab5 via a transcriptional mechanism

**Figure 1:**
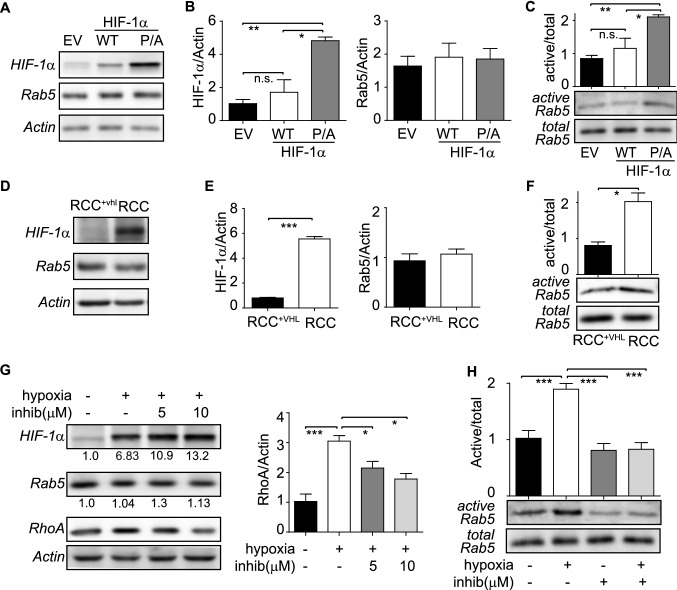
HIF-1α activates Rab5 in normoxia and hypoxia. (**A**–**C**) A549 cells were transfected for 24 h with pcDNA3.1 (EV), pcDNA3.1-HIF-1α^WT^ (WT) or pcDNA3.1-HIF-1α^P402A/P564A^ (P/A) and samples were used for subsequent analysis of total protein expression (**A**,**B**) and Rab5 activity analysis (**C**). (**A**) Representative Western blot images of HIF-1α, Rab5 and actin. (**B**) HIF-1α and Rab5 relative levels were quantified by scanning densitometry analysis and normalized to actin levels. Graphs represent the average from 3 independent experiments (mean ± SEM). *p < 0.05; **p < 0.01; n.s., non-significant. (**C**) Rab5-GTP levels were measured in pulldown assays, as previously described by us (details in materials and methods). Lower panel, representative Western blot images of GTP-bound active Rab5 and total Rab5, respectively. Upper graph shows the average from 3 independent experiments (mean ± SEM), obtained by scanning densitometry analysis of active Rab5 and normalized with respect to total Rab5. *p < 0.05; **p < 0.01; n.s., non-significant. (**D**-**F**) RCC4 and RCC4^+vhl^ cells were grown for 24 h and whole cell lysates were prepared for subsequent analysis of total proteins (**D**, **E**) and Rab5 activity (**F**). (**D**) Representative Western blot images of HIF-1α, Rab5 and actin. (**E**) HIF-1α and Rab5 relative levels were quantified as described in (**B**). Graphs represent the average from 3 independent experiments (mean ± SEM). ***p < 0.001. (**F**) Rab5-GTP levels were measured in pulldown assays, as mentioned in (**C**). Lower panel, representative Western blot images of active and total Rab5. Upper graph shows the average from 3 independent experiments (mean ±  SEM; *p < 0.05). (**G**) A549 cells were incubated in normoxia or hypoxia (1% O_2_) for 24 h, in the presence of either DMSO (vehicle control) or HIF-1α inhibitor (C_26_H_29_NO_5_), at the indicated concentrations, and whole cell lysates were prepared. HIF-1α, Rab5, RhoA and actin levels were assessed by Western blotting and quantified by scanning densitometry analysis. Numerical data below each panel represent relative HIF-1α and Rab5 levels (normalized to actin), respectively, and shown as follows. HIF-1α: normoxia/DMSO (1.00 ± 0.73), hypoxia/DMSO (6.83 ± 2.62), hypoxia/5 μM inhibitor (10.99 ± 2.66), hypoxia/10 μM inhibitor (13.26 ± 4.91). Rab5: normoxia/DMSO (1.00 ± 0.24), hypoxia/DMSO (1.04 ± 0.34), hypoxia/5 μM inhibitor (1.30 ± 0.55), hypoxia/10 μM inhibitor (1.13 ± 0.47). Numerical data are shown as the average of 3 independent experiments (mean ± SEM). The graph indicates relative RhoA levels (normalized to actin), and data are shown as the average of 3 independent experiments (mean ±  SEM). *p < 0.05; ***p < 0.001. (**H**) A549 cells were incubated in normoxia or hypoxia (1% O_2_), in the presence of DMSO or HIF-1α inhibitor, as described in (**G**), and Rab5-GTP levels were determined using the R5BD pulldown assay. Lower panels, representative Western blot images obtained from 3 independent experiments. Upper graph, relative Rab5-GTP levels normalized to total Rab5 by scanning densitometry are shown as the fold increase with respect to normoxia/DMSO. Data represent the average of 3 independent experiments (mean ±  SEM). ***p < 0.001.

Previous studies from our laboratory showed that hypoxia stimulates Rab5 activity (i.e. Rab5-GTP loading), contributing to increased tumor cell migration in vitro and metastasis in vivo^7^. Although those studies suggested a dependency on HIF-1α, as shown by RNAi approaches in hypoxia-challenged cells, the precise role of HIF-1α in Rab5 activation and the mechanisms accounting for such events, remained unexplored. Thus, we first sought to evaluate whether stabilization of HIF-1α under normoxic conditions sufficed to account for Rab5-GTP loading, by transfecting A549 lung carcinoma cells with wild type HIF-1α (HIF-1α^WT^), or a stable, non-hydroxylatable mutant of HIF-1α (HIF-1α^P564A/P402A^). As expected, HIF-1α^P564A/P402A^ accumulated notably upon transfection, whereas HIF-1α^WT^ was increased minimally, when compared with endogenous HIF-1α (Fig. 1A,B). Remarkably, neither the expression of wild type nor of the stabilized HIF-1α mutant altered total Rab5 levels (Fig. 1B), which is consistent with our previous findings showing that hypoxia does not change total Rab5 expression^7^. Most importantly, expression of HIF-1α^P564A/P402A^, but not HIF-1α^WT^, induced a significant increase of Rab5-GTP levels (Fig. 1C), suggesting that the sole stabilization of HIF-1α is sufficient to stimulate Rab5 activity.

These observations were further supported in a cell model of endogenous stabilization of HIF-1α, RCC4 clear cell renal cell carcinoma (CCRCC), which harbor an inactivating mutation in VHL, thereby preventing degradation of endogenous HIF-1α in normoxic conditions^27^. As expected, RCC4 had increased levels of endogenous HIF-1α, when compared with VHL-reconstituted cells (RCC4^+VHL^), and these events were not associated with fluctuations in total Rab5 levels (Fig. 1D,E). However, in accordance with the aforementioned exogenous expression data, endogenous stabilization of HIF-1α (shown here) was associated with augmented Rab5-GTP levels in RCC4 cells (Fig. 1F). Collectively, these data indicate that increased HIF-1α alone suffices to promote Rab5 activation, without changing total Rab5 expression.

Next, we determined whether Rab5-GTP loading depends on HIF-1α-dependent transcription events. To this end, A549 cells were exposed to hypoxia, which is known to stimulate Rab5-GTP loading^7^, and the relevance of HIF-1α activity was evaluated using the small molecule inhibitor, C_26_H_29_NO_5_. As anticipated, C_26_H_29_NO_2_ decreased the expression of HIF-1α target genes in hypoxia, including RhoA and VEGFA (Fig. 1G and data not shown), but not of total Rab5 or hypoxia-stabilized HIF-1α (Fig. 1G). Intriguingly, C_26_H_29_NO_2_ reduced the amount of Rab5-GTP in hypoxia, to levels that were even below those observed in normoxic conditions (Fig. 1H). Taken together, these data indicate that HIF-1α activity is required for Rab5 activation in hypoxia.

### Identification of novel Rab5-GEFs induced by HIF-1α

**Figure 2:**
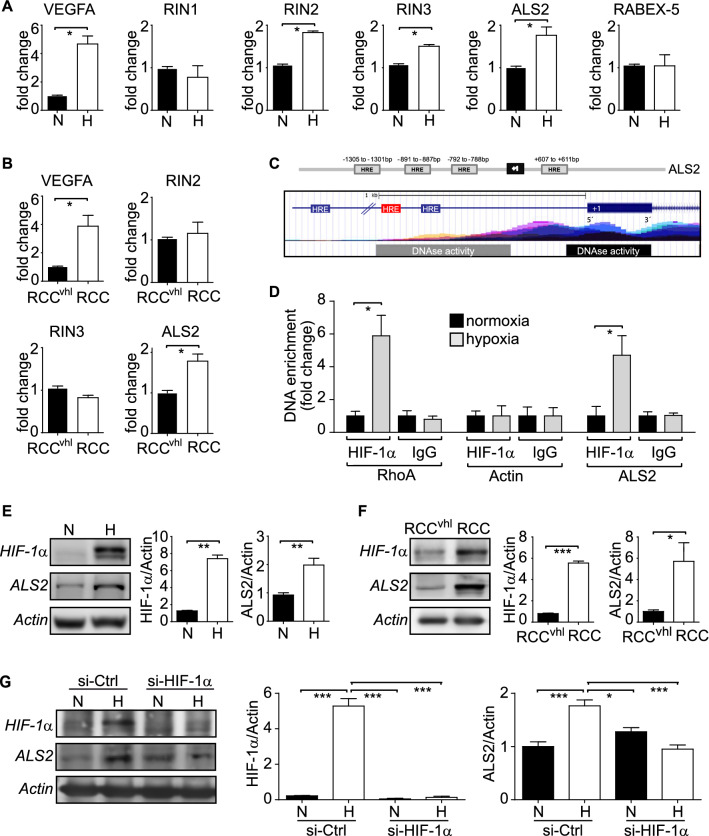
Identification of Rab5GEFs induced by HIF-1α. (**A**) A549 cells were incubated in normoxia (N) and hypoxia (H) for 24 h and total RNA was extracted, for subsequent RT-qPCR analysis of RIN1, RIN2, RIN3, RABEX5 and ALS2. VEGFA was measured as positive control of hypoxia-induced transcription. Relative abundance of all screened Rab5GEFs and VEGFA was normalized to actin, by using the ΔΔCT method, as described in the materials and methods. Graphs show data obtained by averaging 4 independent experiments (mean ±  SEM; *p < 0.05). (**B**) RCC and RCC^+VHL^ cells were grown for 24 h, total RNA was extracted and relative levels of RIN2, RIN3, ALS2 and VEGFA were quantified by RT-qPCR, as described in (**A**). Data are shown as the average of 4 independent experiments (mean ±  SEM; *p < 0.05). (**C**) Upper panel, putative hypoxia response elements (HRE, gray boxes) are shown within the proximal gene promoter of ALS2 (the transcription start site (+ 1) is shown for reference). Lower panel, bioinformatics analysis showing histone acetylation (H3K27ac) marks and DNAse I hypersensitivity within the promoter region of the human genetic sequence of ALS2 (hg38, chromosome 2). H3K27Ac enrichment was obtained from the Chip-Seq database (UCSC Genome Browser Output, hg18) and is shown in mountain/valley color code. Color superposition represents the analysis of at least 7 different cell lines. DNAse I hypersensitivity data was obtained from 95 cell models and shown with respect to intensity and extension. Black rectangle, 95/95 cases; gray rectangle, 52/95 cases. (**D**) Binding of HIF-1α to the proximal promoter of ALS2 was assessed by Chromatin immunoprecipitation (ChIP) assay. HIF-1α was immunoprecipitated with a specific, ChIP-grade antibody in A549 cells exposed to 24 h of normoxia or hypoxia. HSP90 was immunoprecipitated as control (IgG). The enrichment of ALS2 (putative HRE − 792/-788), RhoA (positive control, HRE -1263/-1259, see text for details) and actin was evaluated by qPCR, using specific primers, as described in the materials and methods. The graph shows the quantification of 3 independent experiments (mean ±  SEM; *p < 0.05). (**E**) A549 cells were incubated in normoxia (N) or hypoxia (H) for 24 h and whole cell lysates were prepared for Western blot analysis of HIF-1α, ALS2 and actin. Representative Western blot images are shown, and relative levels of HIF-1α and ALS2 were quantified by scanning densitometry and normalized to actin. Graphs represent the average from 3 independent experiments (mean ±  SEM; **p < 0.01). (**F**) RCC4 and RCC4^+VHL^ cells were grown for 24 h and whole cell lysates were prepared for Western blot analysis of HIF-1α, ALS2 and actin. HIF-1α and ALS2 relative levels were quantified as described in (**E**). Graphs represent the average from 3 independent experiments (mean ±  SEM; *p < 0.05, ***p < 0.001). (**G**) A549 cells were transfected with either siRNA control or mix of 3 different siRNA sequences targeting endogenous HIF-1α for 16 h. Subsequently, cells were incubated in normoxia (N) or hypoxia (H) for an additional 24 h, and whole cell lysates were prepared for Western blot analysis of HIF-1α, ALS2 and actin. Representative Western blot images are shown, and relative levels of HIF-1α and ALS2 were quantified by scanning densitometry and normalized to actin. Graphs represent the average from 3 independent experiments (mean ±  SEM; *p < 0.05, ***p < 0.001).

Since Rab5-GTP loading in hypoxia depends on HIF-1α activity, in the absence of changes in total Rab5 expression, we hypothesized that intermediate, yet to be defined factors, were involved in those events triggered by HIF-1α. In this scenario, we envisioned either, upregulation of guanine nucleotide exchange factors (GEF) or downregulation of a GTPase activating proteins (GAP) as possibilities. Since most studies show that HIF-1α induces, rather than represses gene expression, we focused our screen on putative Rab5-GEFs that could be induced in hypoxia. To this end, Rab5-GEFs that contained putative hypoxia response elements (HRE) within their proximal promoter region (Supplementary Fig. 1A) and that have been associated with the acquisition of malignant cell traits in vitro or are overexpressed in cancer^12–19,28,29^, were selected and analyzed for differential expression in normoxia and hypoxia. This screening procedure narrowed the candidate list down to the Rab5-GEFs RABEX-5, RIN1, RIN2, RIN3 and ALS2. In this group, mRNAs encoding for ALS2, RIN2 and RIN3, but not RABEX-5 or RIN1, increased significantly in hypoxia (Fig. 2A). Importantly, as a positive control for HIF-1α activation, we observed by qPCR that hypoxia induced a fivefold increase in the canonical HIF-1α target gene, VEGFA (Fig. 2A). To validate these observations, we analyzed the expression of these Rab5-GEFs in the RCC4 model of endogenous HIF-1α stabilization, and observed that ALS2, but neither RIN2 or RIN3, increased significantly (Fig. 2B).

Collectively, the previous data suggested that ALS2 is a novel HIF-1α target gene induced by hypoxia. To further assess this possibility, we analyzed the promoter sequence of the ALS2 gene, in search of putative HRE that may account for HIF-1α-dependent induction of ALS2 expression. Albeit we were aware that previous studies had also identified HRE at larger distances upstream (and downstream) of the Transcription Start Site (TSS) of target genes^30^, we operatively limited our primary search for HRE within the first 2 Kb of the ALS2 promoter sequence (defined as ALS2 proximal promoter). This strategy led to the identification of 3 putative HRE (Fig. 2C), among which the HRE located at − 792 bp from the TSS was confirmed as the most likely candidate, based on bioinformatics analysis (UCSC Genome Browser Database) indicating the presence of well-known parameters associated with transcription, including enrichment of histone acetylation (H3K27Ac) and hypersensitivity to DNaseI (Fig. 2C). Hence, functionality of this putative HRE was evaluated in chromatin immunoprecipitation (ChIP) assays. In accordance with our bioinformatics analysis, ChIP data showed enrichment of HIF-1α within the proximal HRE of ALS2 in hypoxia, but not in normoxia (Fig. 2D). Importantly, as a control, the proximal HRE of RhoA, which is known to bind HIF-1α^10^, but not a random sequence within the proximal promoter of the actin gene, was enriched with HIF-1α in hypoxia (Fig. 2D). Taken together, these data indicate that the proximal promoter region of the ALS2 gene contains a functional HRE that binds HIF-1α in hypoxia.

Finally, to evaluate whether transcriptional events triggered by hypoxia and HIF-1α stabilization translate into changes at the protein level, we determined ALS2 levels in hypoxia and in the RCC model of endogenous stabilization of HIF-1α. As anticipated, ALS2 protein levels were increased twofold in hypoxia (Fig. 2E), whereas an almost sixfold increase in ALS2 was detected in RCC4, when compared with RCC4^+VHL^ cells (Fig. 2F). Finally, the requirement of HIF-1α during the hypoxia-driven induction of ALS2 was assessed by siRNA targeting of endogenous HIF-1α in hypoxia. As expected, targeting of endogenous HIF-1α abrogated its stabilization in hypoxia and, most importantly, ALS2 induction in hypoxia was prevented by siRNA-mediated downregulation of HIF-1α (Fig. 2G). These data indicate that HIF-1α stabilization is required for the induction of ALS2 expression in hypoxia.

### ALS2 is required for hypoxia-driven Rab5 activation, tumor cell migration and invasion

**Figure 3:**
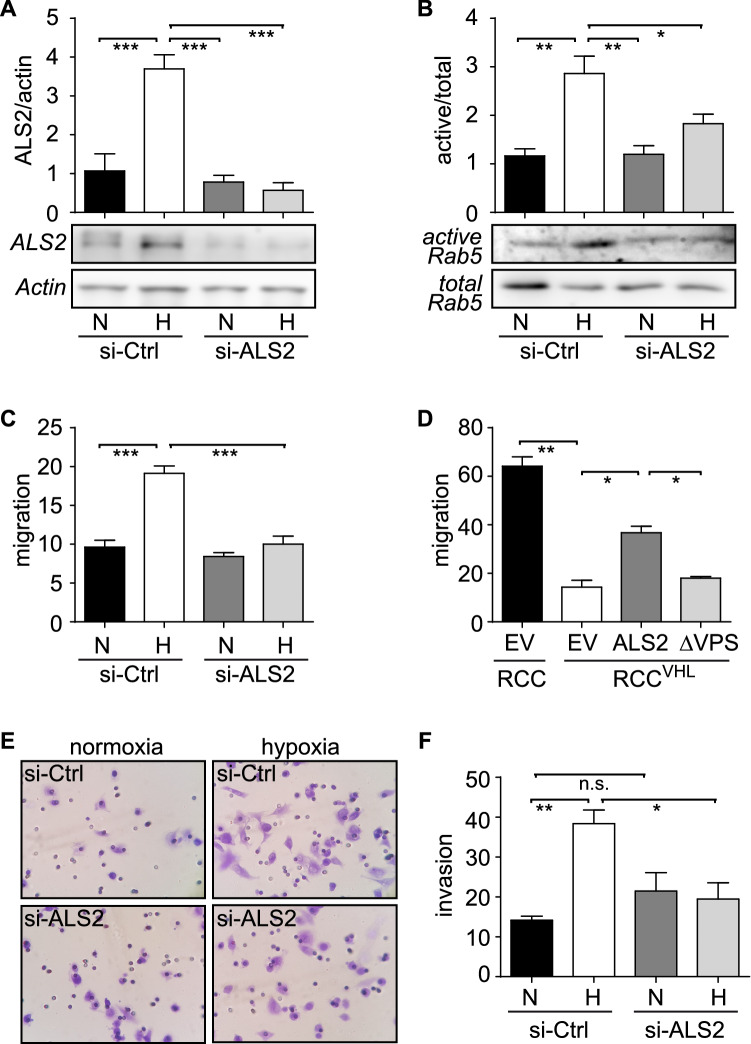
ALS2 is required for hypoxia induced Rab5 activity, tumor cell migration and invasion. (**A**–**C**) A549 cells were transfected with control (si-Ctrl) or ALS2 (si-ALS2) targeting siRNA constructs, exposed to normoxia (N) or hypoxia (H) for 24 h and used for subsequent analysis. **(A)** Whole cell lysates were prepared and analyzed by Western blotting with antibodies against ALS2 and actin. Representative images are shown and relative levels of ALS2 were quantified by scanning densitometry and normalized to actin. Graph represents the average from 3 independent experiments (mean ±  SEM; *** *p* < 0.001). (**B**) Rab5-GTP levels were measured by R5BD pulldown assays. Lower panel, representative Western blot images of GTP-bound active Rab5 and total Rab5, respectively. Upper graph shows the average from 3 independent experiments, obtained by scanning densitometry analysis of active Rab5 and normalized with respect to total Rab5 (mean ±  SEM; *p < 0.05; **p < 0.01). (**C**) Cell migration was assessed in Boyden Chambers, as previously standardized by us (for details, see materials and methods). Cells were allowed to migrate for 2 h in Transwell chambers, coated with 2 μg/ml fibronectin, under normoxic conditions. Cells that migrated were visualized by crystal violet staining. Data represent the average from 3 independent experiments (mean ±  SEM; ***p < 0.001). (**D**) RCC4 and RCC4^+VHL^ cells were transfected for 24 h with either empty vector control (GFP), full-length ALS2 (GFP-ALS2^WT^) or VPS9-deficient ALS2 (ALS2^ΔVPS9^). Cells were harvested and used for subsequent cell migration analysis. Data represent the average from 3 independent experiments (mean ±  SEM; *p < 0.05). (**E**, **F**) A549 cells were transfected for 24 h with control or ALS2 targeting siRNA constructs, harvested, seeded onto Matrigel Chambers and allowed to invade in normoxia (N) or hypoxia (H) for 24 h. Representative images are shown. Graph represents the quantification of 3 independent experiments (mean ±  SEM; *p < 0.05; **p < 0.01; n.s., non-significant).

Hypoxia is known to promote tumor cell migration, invasion and metastasis (reviewed in^1^) and our recent studies showed that increased Rab5-GTP loading in hypoxia is involved in these events^7^. Hence, the identification of the Rab5GEF ALS2 as a HIF-1α target gene prompted us to evaluate the impact of ALS2 induction in tumor cell migration, invasion and metastasis. First, we assessed the requirement of ALS2 for Rab5-GTP loading in hypoxia, by targeting endogenous ALS2 with a siRNA-based approach. As anticipated, hypoxia provoked a significant increase of endogenous ALS2 in siRNA-control, but not siRNA-ALS2 cells (Fig. 3A). Most importantly, siRNA-mediated targeting of endogenous ALS2 prevented Rab5-GTP loading induced by hypoxia (Fig. 3B). In line with these observations and with reports showing a requirement for Rab5 in tumor cell migration, ALS2 was necessary, because ALS2 downregulation prevented cell migration in hypoxia (Fig. 3C). Accordingly, decreased ALS2 levels in RCC4^+VHL^ cells were associated with reduced cell migration, when compared with RCC4 cells. Alternatively, transfection of RCC^+VHL^ cells with GFP-ALS2, but not GFP alone, resulted in partial recovery of cell migration in this model (Fig. 3D). To determine whether the effects of ALS2 on cell migration induced by hypoxia and HIF-1α were due to its GEF activity towards Rab5, we used a VPS-deficient mutant of ALS2 (GFP-ALS2^ΔVPS^), previously shown to lack Rab5-GEF activity^22^. It was found that, unlike wild type ALS2, expression of VPS-deficient ALS2 failed to promote migration of RCC^+VHL^ cells (Fig. 3D). Taken together, these data indicate that ALS2 is required for hypoxia-induced cell migration and that these events depend on its GEF activity towards Rab5.

**Figure 4:**
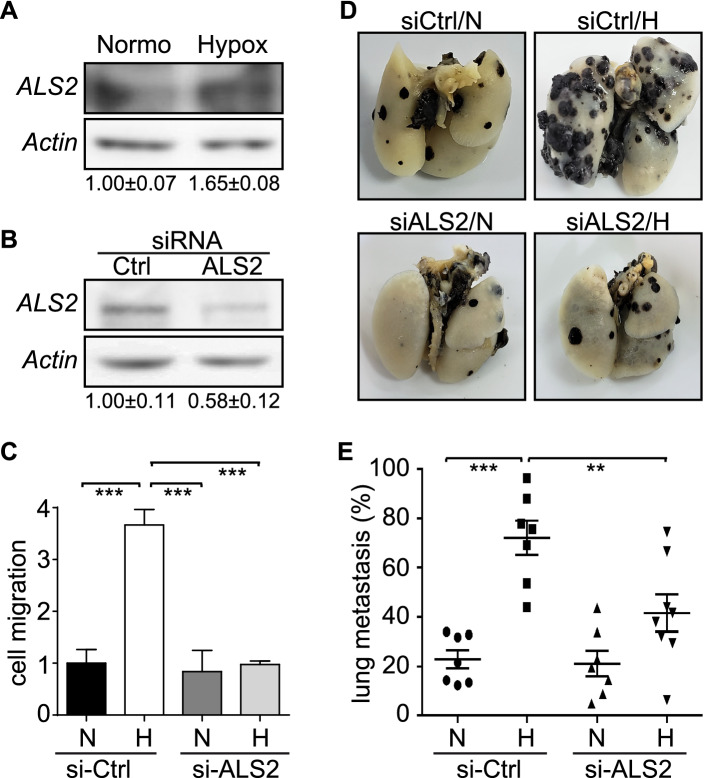
ALS2 participates in hypoxia-induced melanoma cell migration in vitro and lung colonization in vivo. (**A**) B16-F10 murine melanoma cells were incubated in normoxia or hypoxia for 24 h and whole cell lysates were prepared for Western blot analysis of ALS2 and actin. Representative Western blot images are shown, and relative levels of ALS2 were quantified by scanning densitometry and normalized to actin. Numbers below each panel indicate the average from 3 independent experiments (mean ± SEM). (**B**) B16-F10 cells were transfected with either control siRNA or a mix of siRNA sequences targeting murine ALS2 (for full description, see materials and methods) and whole cell lysates were prepared at 48 h post-transfection. Representative Western blot images are shown for ALS2 and Actin, and numerical data below panels represent the average from three independent experiments (mean ± SEM). (**C**) B16-F10 cells were transfected with either control siRNA or a mix of siRNA sequences targeting murine ALS2, and simultaneously exposed to normoxia (N) or hypoxia (H) for 24 h. Cells were then harvested and allowed to migrate for 2 h in Transwell chambers, coated with 2 μg/ml fibronectin, under normoxic conditions. Cells that migrated were visualized by crystal violet staining. Data represent the average from 3 independent experiments (mean ±  SEM; ***p < 0.001). (**D**,**E**) B16-F10 cells were transfected with either control siRNA or a mix of siRNA sequences targeting murine ALS2, and simultaneously exposed to normoxia (N) or hypoxia (H) for 24 h. Cells were then harvested, re-suspended in physiological saline (2 × 10^5^ cells) and injected intravenously into the tail vein of C57BL/6 mice. Black lung tumor mass due to colonization was quantified after sacrificing the animals at day 21 post-injection. (**D**) Representative images are shown. (**E**) Quantification of lung tumor mass was performed as detailed in the materials and methods. Results are shown for 7 mice (siRNA-control/N, siRNA-control/H, siRNA-ALS2/N) or 8 mice per group (siRNA-ALS2/H). N, normoxia; H, hypoxia (**p < 0.01, ***p < 0.001).

Next, we evaluated whether the effects of ALS2 in cell migration induced by hypoxia translated into responses that contribute to tumor cell invasiveness and metastasis in vivo. Indeed, ALS2 was found to be required for hypoxia-induced invasion, as shown by siRNA-mediated targeting of endogenous ALS2 in A549 cells (Fig. 3E,F). To extend these observations to the preclinical level, we used B16-F10 mouse melanoma cells in the isogenic model of experimental metastasis (lung colonization assay) in C57BL/6 mice, as previously described by our group^7^. First, we assessed whether hypoxia induced ALS2 expression and then whether there was a requirement for ALS2 in hypoxia-induced migration of B16-F10 cells. As observed for A549 and RCC cells, hypoxia caused a significant increase in ALS2 levels in B16-F10 cells (Fig. 4A). Thereafter, B16-F10 cells were transiently transfected with either control siRNA or a mix of siRNAs targeting murine ALS2, resulting in a substantial reduction of ALS2 expression after 48 h (Fig. 4B) and abrogation of hypoxia-induced cell migration (Fig. 4C). Of note, as a control, downregulation of ALS2 in B16-F10 cells neither affected cell viability in normoxia or hypoxia (Supplementary Fig. 1B). B16-F10 cells were transiently transfected with siRNA-control or siRNA-ALS2 constructs and simultaneously exposed to normoxia or hypoxia for 24 h, then injected into the tail vein of C57BL/6 mice, and lung colonization was evaluated 21 days later, as previously reported^7,25^. In accordance with our previous observations^7^, hypoxia augmented the extent of lung colonization in siRNA-control cells; however, knockdown of endogenous ALS2 resulted in a significant decrease in hypoxia-induced lung colonization (Fig. 4D,E). Taken together, these data indicate that ALS2 contributes to tumor cell invasion and experimental metastasis induced by hypoxia.

### ALS2 is overexpressed in cancer

**Figure 5:**
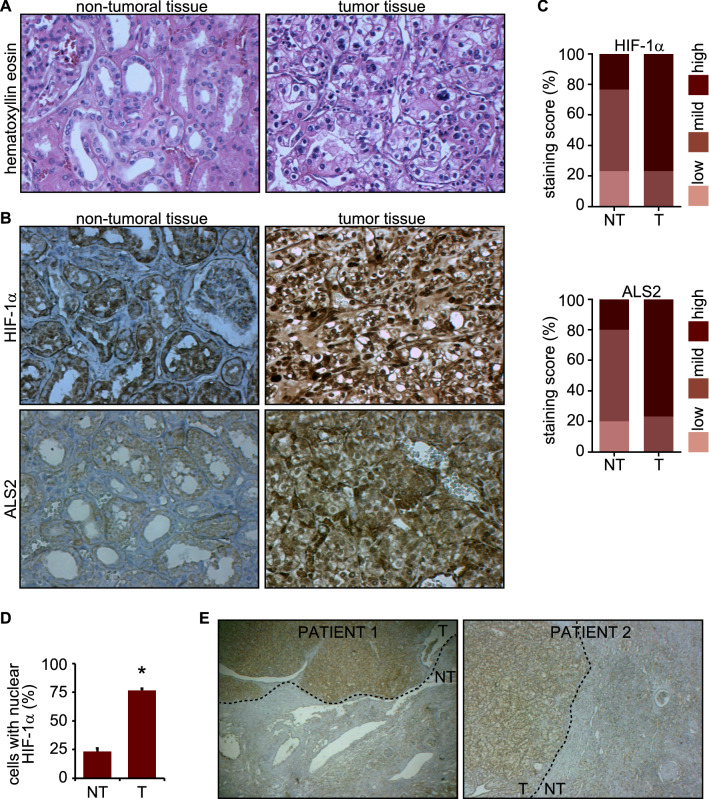
ALS2 is overexpressed in clear cell renal cell carcinoma biopsies. Biopsies with diagnosis of clear cell renal cell carcinoma and tumor free peritumoral region were used for histochemical analyses. (**A**) Representative images of hematoxylin eosin staining. (**B**) Tissue sections were incubated with primary antibodies against HIF-1α or ALS2, then incubated with peroxidase-streptavidin conjugates and visualized with diaminobenzidine, contrasted with Harry´s hematoxylin. (**C**) Percentages of staining scores (low, mild or high expression) for HIF-1α and ALS2 in tumor (T) and non-tumoral (NT) tissues. CCRCC samples, n = 30; and non-tumoral samples, n = 30 (data are summarized in Supplementary Fig. 3). (**D**) Percentage of cells with nuclear localization of HIF-1α, obtained from images as shown in (**B**). Quantifications were obtained as described in the materials and methods (mean ±  SEM; *t* test; ***p ≤ 0.001). (**E**) Two different cases depicting tumor (T) and non-tumor (NT) regions within the same sample are shown.

Collectively, our in vitro and in vivo data identified a causal relationship between hypoxia, HIF-1α stabilization and ALS2 expression. To evaluate the relevance of this association in cancer, we assessed the expression of ALS2 in clear cell renal cell carcinoma (CCRCC) biopsies, since this type of cancer is associated with mutations in VHL^6^. Immunohistochemical analysis showed higher levels of HIF-1α in CCRCC, when compared with non-tumor samples (Fig. 5A–C, Supplementary Fig. 3A). Additionally, substantial accumulation of nuclear HIF-1α was observed in CCRCC, but not in non-tumor biopsies (Fig. 5D). Remarkably, although ALS2 was detected in both non-tumor and CCRCC samples, levels of ALS2 were substantially higher in the tumors (Fig. 5B,C and Supplementary Fig. 3B). In fact, ALS2 levels fluctuated within the same biopsies, where high ALS2 detection was observed within the tumor area, but not in the normal adjacent region (Fig. 5E). Immunohistochemical analysis showed a strong association between the expression of ALS2 and HIF-1α, with a Spearman’s Correlation Coefficient of 0.728 (*p* = 0.03; n = 30 cases). Taken together, these data indicate that ALS2 is upregulated in CCRCC samples, and that the expression of ALS2 is strongly associated with the stabilization of HIF-1α. Finally, database screening showed that augmented ALS2 expression correlates with poor patient survival in a subgroup of cancers, including adrenocortical carcinoma, papillary renal cell carcinoma and lung squamous cell carcinoma, although contrasting results were found in others, such as leukemia and CCRCC (Supplementary Fig. 4). Collectively, these data indicate that ALS2 expression generally coincides with the presence of HIF-1α, but that ALS2 upregulation is associated with poor prognosis in a subgroup of cancers.

## Discussion

**Figure 6:**
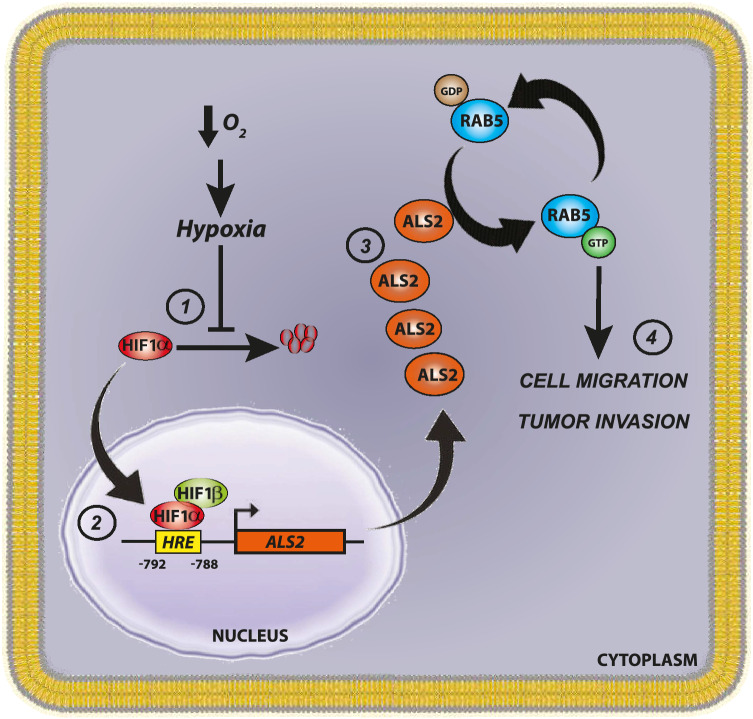
Proposed model. In hypoxia, HIF-1α stabilizes and heterodimerizes with HIF-1β allowing the transcription of ALS2 via binding to the HRE located within the proximal promoter (− 792 to − 788) of ALS2. The latter leads to increased ALS2 protein expression, allowing Rab5-GTP loading, enhanced tumor cell migration, invasion and metastasis.

This study identifies a novel hypoxia-inducible target gene, ALS2, which is overexpressed in a HIF-1α-related malignancy and promotes the acquisition of aggressive traits in tumor cells. Upregulation of ALS2 in hypoxia accounted for increased Rab5-GTP loading, thereby leading to the activation of downstream pathways, that depend on hypoxia and are involved in tumor cell migration, invasion and experimental metastasis (Fig. 6, proposed model). Specifically, we identified ALS2 as a HIF-1α target gene, based on promoter binding data, bioinformatics analysis, interference assays and transfection/recovery experiments in different cellular settings. It should be noted that 7 GEFs have been described to activate Rab5, namely ALS2, RIN1, RIN2, RIN3, RABEX-5, ALS2CL and RME-6^31,32^. In this study, we evaluated 5 Rab5-GEFs, on the basis of criteria that included: (1) presence of putative hypoxia response elements (HRE) within the proximal promoter region (up to 2 Kb upstream of the TSS) of genes encoding for these RabGEFs (Supplementary Fig. 1); (2) their involvement in aspects related to malignancy or cancer progression. Following these criteria, the analysis did not include ALSCL or RME-6, and hence, we cannot exclude the possibility that these, or other GEFs yet to be identified, may represent additional targets of HIF-1α.

ALS2 is known to control several aspects of the endocytic trafficking via promoting Rab5 activation, although most of evidence has been provided in neuronal cell models^33–37^. Conversely, a few studies have investigated the role of ALS2 in non-neuronal cell types, and most importantly, our understanding the effects of ALS2 in cellular aspects that are beyond those reported in neuronal function, remain limiting. Specifically, using COS-7 and HeLa cells, ALS2 was shown to promote Rab5 activation at macropinosomes^38^, whereas mutant ALS2 failed to localize to macropinosomes and was associated with defective autophagy/endolysosomal dynamics in HeLa cells^39^ and in ALS2-deficient primary cultured neurons^40^. Likewise, in fibroblasts, ALS2 was reported to co-localize with Rac1 at membrane ruffles and peripheral lamellipodia, suggesting a role in endocytic trafficking and cytoskeleton remodeling^22^. Subsequent studies showed that ALS2 is recruited by activated Rac1 to membrane ruffles, increasing macropinocytosis and contributing to their fusion with early endosomes via Rab5^41^. Collectively, all these observations are intriguing, since it is known that Rab5-dependent macropinocytosis and cytoskeleton remodeling via Rac1 activation are critical events in tumor cell migration, as shown in HeLa, A375m2 melanoma and BE colon carcinoma cells^42^. Therefore, the possibility that ALS2 modulates tumor cell migration via activation of Rab5 was attractive. Indeed, we showed here that ALS2 is required for (hypoxic) stimuli-induced migration of tumor cells, as observed in renal cell carcinoma, murine melanoma and lung carcinoma cell lines.

Another challenging possibility that emerges from these observations is the potential link between hypoxia and tumor cell viability via ALS2-dependent activation of “a salvage pathway”. The latter idea is supported by recent studies showing that oxidative stress-induced cytotoxicity in HeLa cells is prevented via an unanticipated mechanism involving translocation of Rab5 from early endosomes to the mitochondria (at specific mitochondria/endosome contact sites), where it is activated by ALS2, thereby preventing cytochrome C release, oxygen consumption and cell death. Consequently, ALS2 deficiency was associated with increased cytotoxicity^19^. It is thus tempting to speculate that ALS2 induction in hypoxia may constitute a novel “salvage pathway” in tumor cells exposed to hypoxia. However, our data obtained in B16-F10 melanoma cells indicate that neither ALS2 knockdown nor 24 h hypoxia affected cell viability. Future studies will be required to address these intriguing possibilities.

It is interesting that, although several Rab5GEFs, including ALS2, RIN2 and RIN3, were found to be upregulated in hypoxia, only ALS2 depended on HIF-1α stabilization, as shown in transfection, siRNA and endogenous stabilization (VHL mutation) models. This suggests that the remaining Rab5GEFs, RIN2 and RIN3, respond to hypoxia independently of HIF-1α stabilization. In this context, it is possible that additional, yet to be identified factors activated in hypoxia, such as reactive oxygen species or the metabolic sensor PGC-1α (peroxisome-proliferator-activated receptor-γ coactivator-1α)^43–45^, contribute to the induction of other Rab5GEFs. Unlike the remaining Rab5GEFs, ALS2 was identified as a HIF-1α target gene with a functional HRE within the proximal promoter region (site − 788 bp), which is associated with parameters of active transcription, including DNase I hypersensitivity and H3K27ac enrichment. Of note, although not shown here, this newly identified HRE is flanked by conserved putative auxiliary sequences, previously suggested to be necessary for the activity of HIF-1α^46,47^. However, it is worth mentioning that other putative HREs, contained within this proximal promoter region of the ALS2 gene (Fig. 2) were not evaluated here. Similarly, it has been reported that HRE may lay further upstream or downstream the TSS of HIF-1α target genes^30^. Hence, because HIF-1α target genes can harbor more than one functional HRE, we cannot exclude the involvement of additional HRE in HIF-1α-dependent induction of ALS2. This important aspect must be addressed in future studies.

Finally, these in vitro observations were shown to be of clinical relevance, given that ALS2 was found upregulated in CCRCC samples and that correlated with the stabilization and nuclear localization of HIF-1α. Of note, these immunohistochemical data agree with previous studies, showing only modest expression of ALS2 in healthy renal tissue^36^. Therefore, the findings reported here will be relevant to understand the effects of hypoxia in patient prognosis, favoring tumor cell metastasis and resistance to chemotherapy. In conclusion, this study uncovers the Rab5GEF ALS2 as a novel hypoxia inducible target gene that is associated with augmented tumor cell migration, invasion and metastasis (Fig. 6).

## Supplementary Information

Supplementary Information
