# Supplementary material for: The RabGEF ALS2 is a hypoxia inducible target associated with the acquisition of aggressive traits in tumor cells
Source: Sci Rep. 2020 Dec 18;10:22302. doi: 10.1038/s41598-020-79270-6 (PMC7749157; doi:10.1038/s41598-020-79270-6)

The RabGEF ALS2 is a hypoxia inducible target associated with the acquisition of aggressive traits in tumor cells

Solange Rivas[1,2], Patricio Silva[1,2], Montserrat Reyes[3], Hugo Sepúlveda[4], Luis Solano[1,2], Juan Acuña[5], Marisol Guerrero[5], Manuel Varas-Godoy[6], Andrew F.G. Quest[2,7], Martín Montecino[4] and Vicente A. Torres[1,2#]

[1]*Institute for Research in Dental Sciences, Faculty of Dentistry, Universidad de Chile, Santiago, Chile*
[2]*Advanced Center for Chronic Diseases (ACCDiS), Universidad de Chile, Santiago, Chile*
[3]*Department of Pathology and Oral Medicine, Faculty of Dentistry, Universidad de Chile, Santiago, Chile*
[4]*Institute of Biomedical Sciences and FONDAP Center for Genome Regulation, Faculty of Medicine and Faculty of Life Sciences, Universidad Andrés Bello, Santiago, Chile*
[5]*Laboratory of Pathological Anatomy, Hospital San José, Santiago, Chile*
[6]*Center for Cell Biology and Biomedicine (CEBICEM), Faculty of Medicine and Science, Universidad San Sebastián, Santiago, Chile*
[7]*Center for Studies on Exercise, Metabolism and Cancer (CEMC), Biomedical Sciences Institute (ICBM), Faculty of Medicine, Universidad de Chile, Santiago, Chile*

The RabGEF ALS2 is a hypoxia inducible target associated with the acquisition of aggressive traits in tumor cells.

Solange Rivas, Patricio Silva, Montserrat Reyes, Hugo Sepúlveda, Luis Solano, Juan Acuña, Marisol Guerrero, Manuel Varas-Godoy, Andrew F.G. Quest, Martín Montecino and Vicente A. Torres

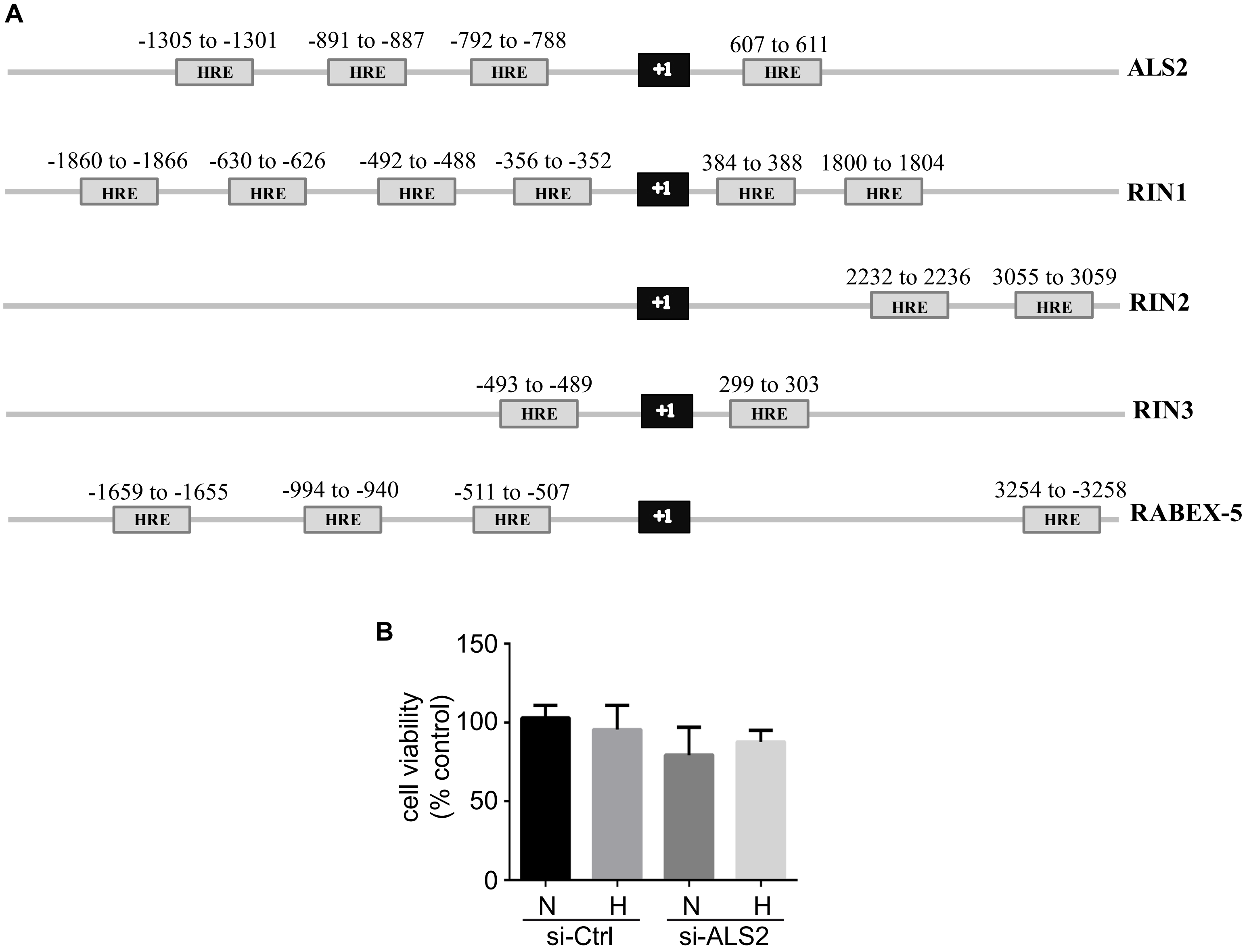

Supplementary Figure 1. (A) In silico analysis of putative HIF-1α target genes. Complete gene sequences of different Rab5GEFs were obtained from http://www.ensembl.org/Homo_sapiens/Gene/ database. Specifically, the proximal promoter regions of ALS2 (ENSG00000003393), RIN1 (ENSG00000174791), RIN2 (ENSG00000132669), RIN3 (ENSG00000100599), RABEX-5 (ENSG00000154710), were defined in accordance with their nucleotide sequence up to -2000 bp. The +1 site and putative consensus hypoxia response elements (HRE, 5'-[A/G]CGTG-3´) are shown within these sequences. **(B, C)** *In vitro* transfection experiments in B16-F10 mouse melanoma cells. Cells were transfected with either control siRNA or a mix of siRNA sequences targeting murine ALS2 and simultaneously exposed to 24 h of normoxia (N) or hypoxia (H) for subsequent analysis. Cell viability was measured with the trypan blue exclusion assay. Data were averaged from 3 independent experiments and shown as the percentage of cell viability relative to cells transfected with siRNA-control in normoxia (mean ± s.e.m).

The RabGEF ALS2 is a hypoxia inducible target associated with the acquisition of aggressive traits in tumor cells.

Solange Rivas, Patricio Silva, Montserrat Reyes, Hugo Sepúlveda, Luis Solano, Juan Acuña, Marisol Guerrero, Manuel Varas-Godoy, Andrew F.G. Quest, Martín Montecino and Vicente A. Torres

Antibody	Sample	Tested Dilution	Optimal Dilution	Incubation Time
HIF-1α	Oral squamous cell carcinoma	1:50-1:500	1:100	1 hour 37°C
	Clear cell renal cell carcinoma	1:50-1:500	1:100	1 hour 37°C
	Healthy oral mucosa	1:50-1:500	1:100	1 hour 37°C
	Healthy kidney tissue	1:50-1:500	1:100	1 hour 37°C
ALS2	Breast cancer	1:50-1:200	1:50	1 hour 37°C
	Healthy kidney tissue	1:50-1:200	1:50	1 hour 37°C

Supplementary Figure 2. Standardization of HIF-1α and ALS2 antibodies for immunohistochemistry. Antibodies were standardized according to the manufacturer's recommendations in different tissues, including oral squamous cell carcinoma, clear cell renal cell carcinoma, healthy oral mucosa and non-tumor renal tissue. Breast cancer samples were included for ALS2 standardization. Different concentrations of each antibody were tested by incubation at 37°C, 1 h, and optimal dilutions were chosen and used for study samples.

The RabGEF ALS2 is a hypoxia inducible target associated with the acquisition of aggressive traits in tumor cells.

Solange Rivas, Patricio Silva, Montserrat Reyes, Hugo Sepúlveda, Luis Solano, Juan Acuña, Marisol Guerrero, Manuel Varas-Godoy, Andrew F.G. Quest, Martín Montecino and Vicente A. Torres

A

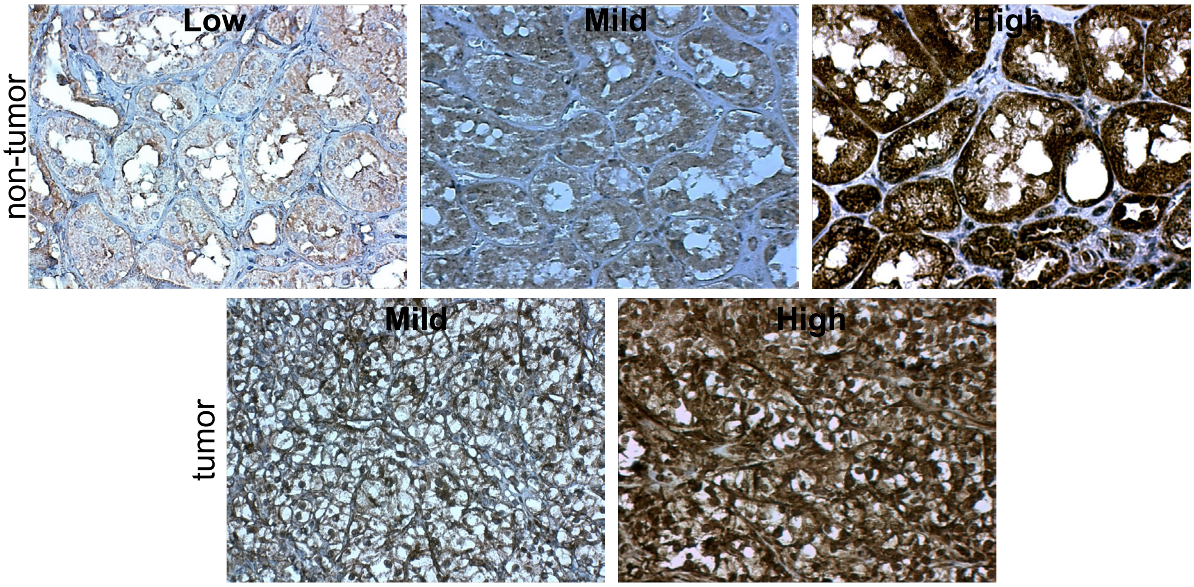

Groups	Total cases	Intensity of staining HIF-1α			Localization of HIF-1α	
		low	mild	high	cytoplasmic	nuclear
Non-tumor tissue	30	7 (23.3%)	16 (53.3%)	7 (23.3%)	30 (100%)	7 (23.3%)
Tumor tissue	30	0 (0%)	7 (23.3%)	23 (76.6%)	30 (100%)	23 (76.6%)

B

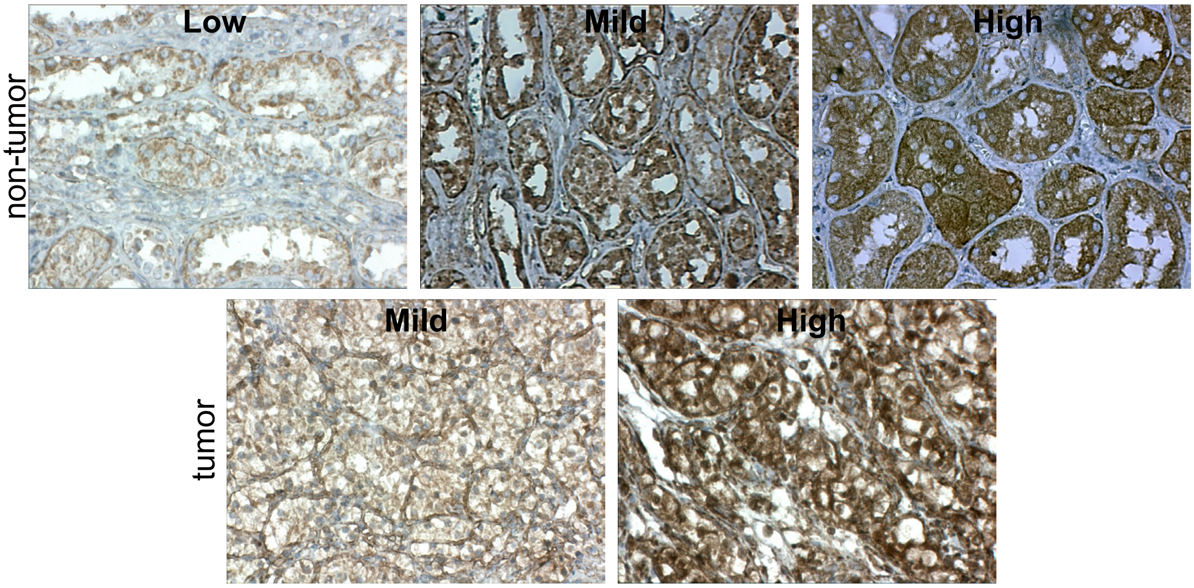

Groups	Total cases	Intensity of staining ALS2			Localization of ALS2	
		low	mild	high	cytoplasmic	nuclear
Non-tumor tissue	30	6 (20%)	18 (60%)	6 (23%)	30 (100%)	2 (6.6%)
Tumor tissue	30	0 (0%)	7 (23.3%)	23 (76.6%)	30 (100%)	29 (96.6%)

Supplementary Figure 3. Percentage of staining scores (low, mild or high expression) for HIF-1α **(A)** and ALS2 **(B)** in tumor and non-tumoral tissues. Intensity scores and subcellular localization (nuclear and/or cytoplasmic) are shown in the tables (tumor tissue, n=30; non-tumor tissue n=30). Representative images are shown for HIF-1α **(A)** and ALS2 **(B).**

The RabGEF ALS2 is a hypoxia inducible target associated with the acquisition of aggressive traits in tumor cells.

Solange Rivas, Patricio Silva, Montserrat Reyes, Hugo Sepúlveda, Luis Solano, Juan Acuña, Marisol Guerrero, Manuel Varas-Godoy, Andrew F.G. Quest, Martín Montecino and Vicente A. Torres

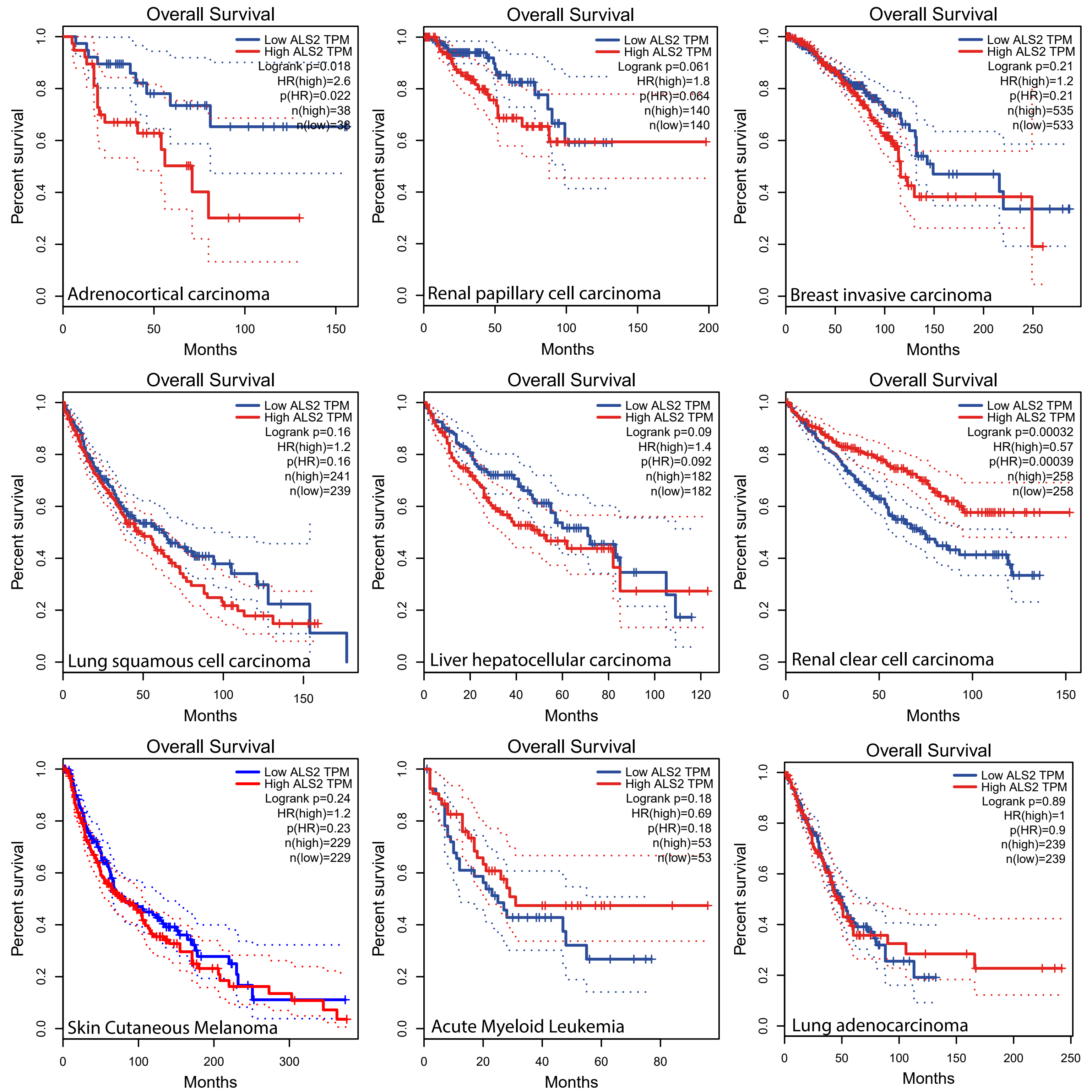

Supplementary Figure 4. Kaplan-Meier analyses were performed in different cancers, by using the GEPIA web server (gepia.cancer-pku.cn). Each graph shows overall survival of patients with high (red) or low (blue) expression of ALS2 in different cancers, including adrenocortical carcinoma, renal papillary cell carcinoma, breast invasive carcinoma, lung squamous cell carcinoma, liver hepatocellular carcinoma, clear cell renal cell carcinoma, skin cutaneous melanoma, acute myeloid leukemia and lung adenocarcinoma. The median of TPM (transcript per million) was used to separate high or low expression of ALS2. The inset shows the p value for log-rank test (Mantel-Cox test) (*p <0,05); Hazard ratio (HR) related to high expression curve and its p value (*p <0.05); n=samples.

The RabGEF ALS2 is a hypoxia inducible target associated with the acquisition of aggressive traits in tumor cells.

Solange Rivas, Patricio Silva, Montserrat Reyes, Hugo Sepúlveda, Luis Solano, Juan Acuña, Marisol Guerrero, Manuel Varas-Godoy, Andrew F.G. Quest, Martín Montecino and Vicente A. Torres

Supplementary Figure 5

Figure 1A

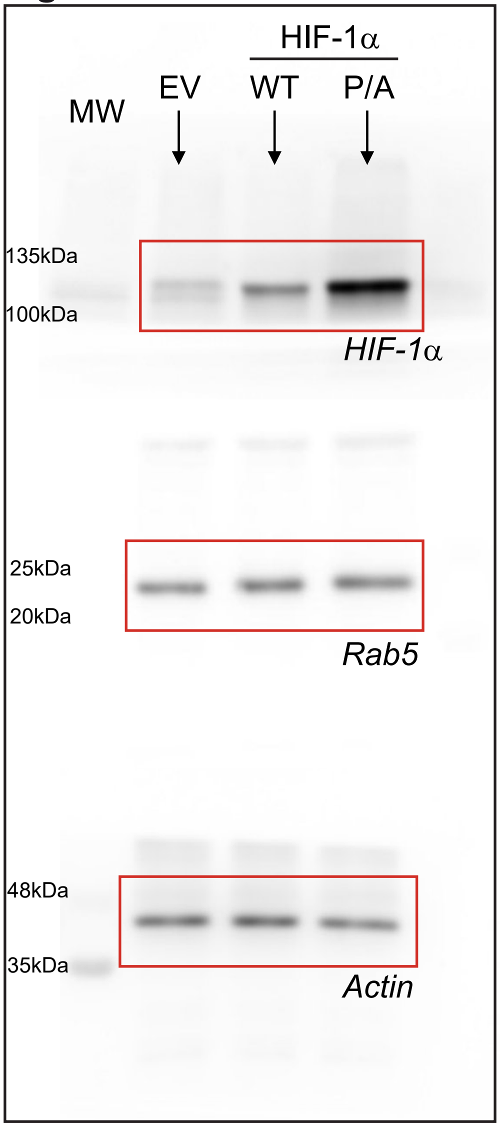

Figure 1D

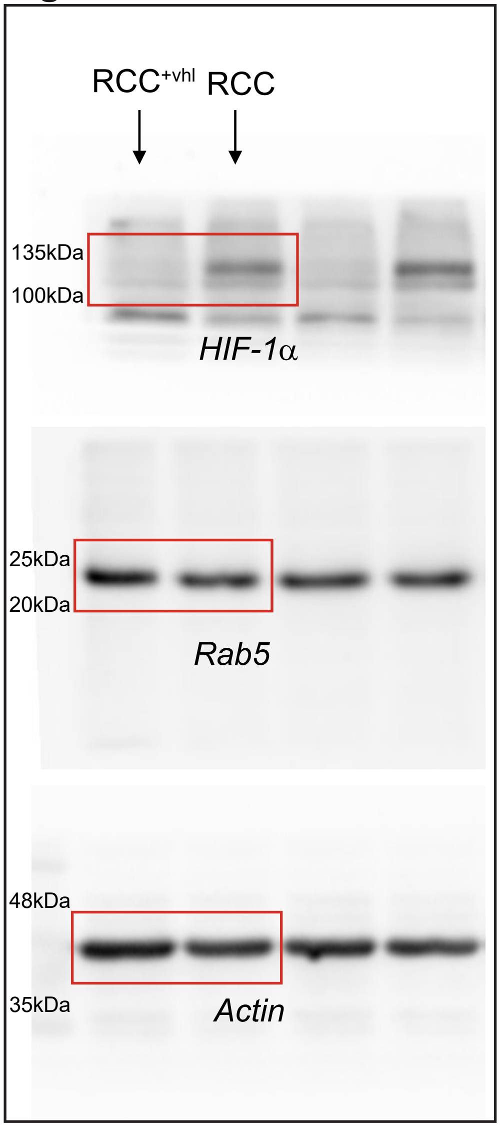

Figure 1C

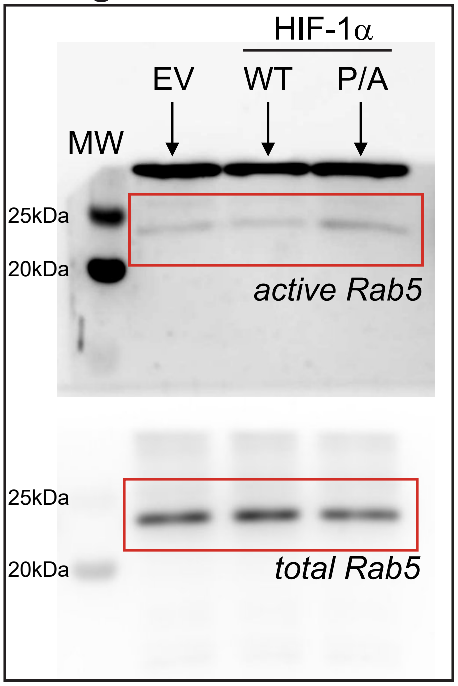

Figure 1G

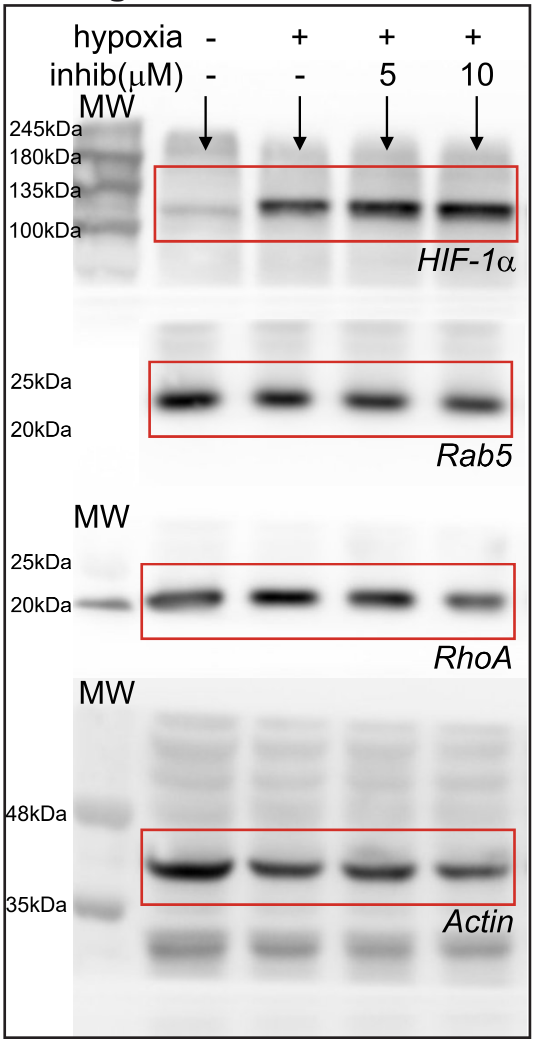

Figure 1F

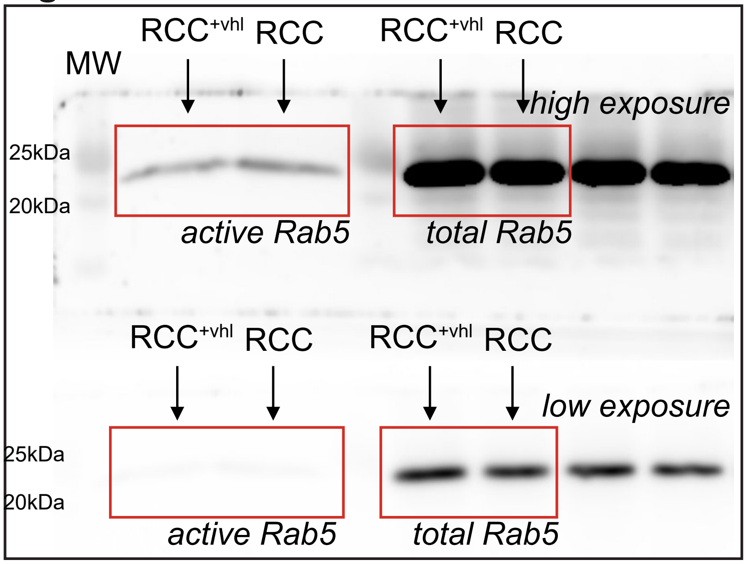

The RabGEF ALS2 is a hypoxia inducible target associated with the acquisition of aggressive traits in tumor cells.

Solange Rivas, Patricio Silva, Montserrat Reyes, Hugo Sepúlveda, Luis Solano, Juan Acuña, Marisol Guerrero, Manuel Varas-Godoy, Andrew F.G. Quest, Martín Montecino and Vicente A. Torres

Supplementary Figure 6

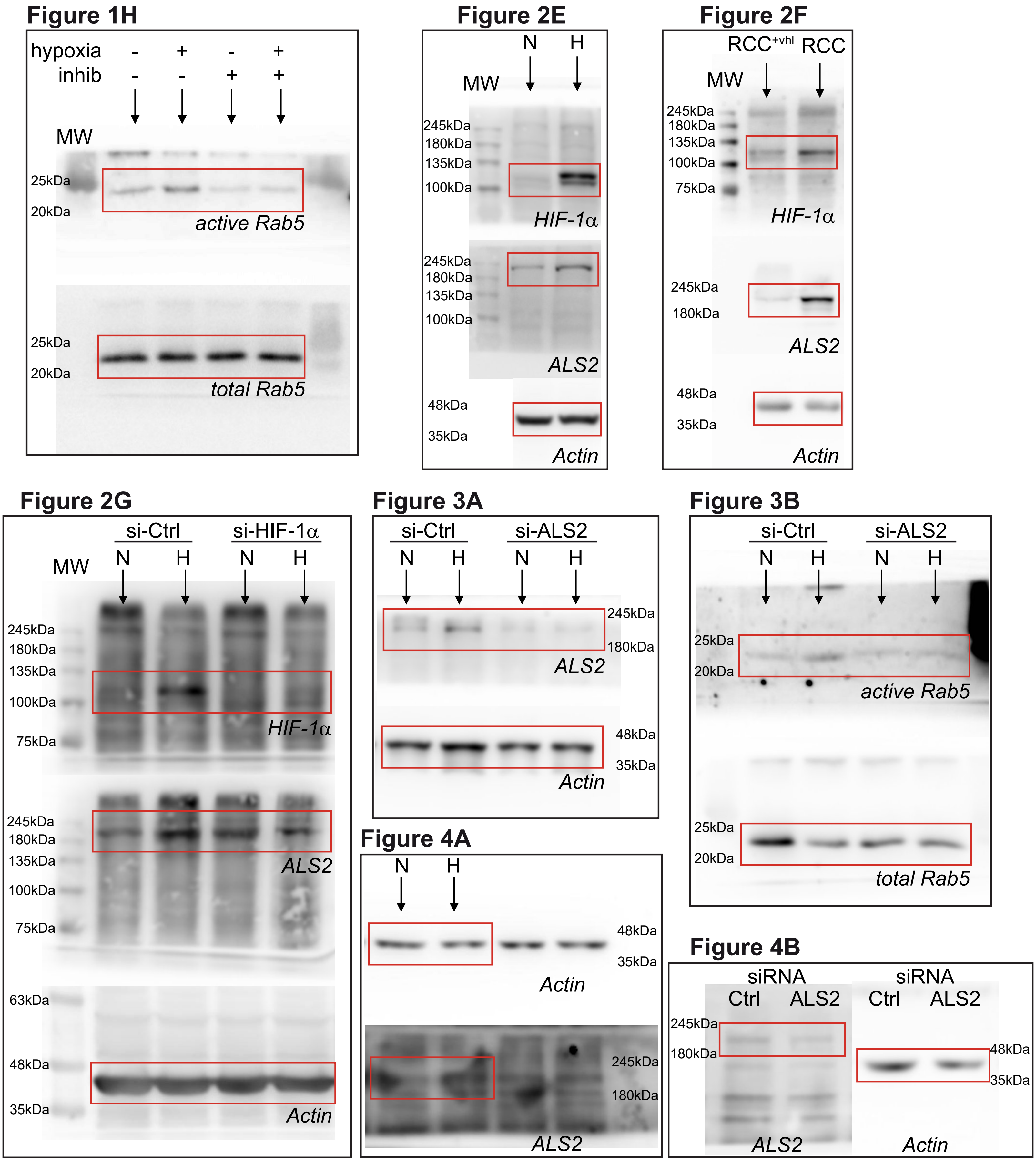

Supplement: Supplementary file 1 — Supplementary Information [file 41598_2020_79270_MOESM1_ESM.pdf]
